# Unveiling the Cultivation of *Nostoc* sp. under Controlled Laboratory Conditions

**DOI:** 10.3390/biology13050306

**Published:** 2024-04-28

**Authors:** Teresa Mouga, Jéssica Pereira, Vitória Moreira, Clélia Afonso

**Affiliations:** 1 MARE-Marine and Environment Research Center/ARNET-Aquatic Research Network, School of Tourism and Maritime Technology, Polytechnic University of Leiria, 2520-614 Peniche, Portugal; clelia@ipleiria.pt; 2 School of Tourism and Maritime Technology, Polytechnic University of Leiria, 2520-614 Peniche, Portugal

**Keywords:** *Nostoc* sp. 136, initial density, nutrient supplementation, irradiance, specific growth rate, productivity

## Abstract

**Simple Summary:**

The study focused on cultivating the cyanobacterial strain *Nostoc* sp. 136 under controlled laboratory conditions. Various factors such as nutrient media, initial biomass concentration, nutrient supplementation, and light conditions were investigated to optimize growth and productivity. Results showed that the strain adapted well to the laboratory conditions, with the highest growth rates observed at lower initial biomass concentrations and with mBG11 medium. Nutrient supplementation, particularly nitrogen, showed potential for enhancing growth, while different light wavelengths had varied effects on growth and productivity. Biochemical analysis revealed promising levels of proteins and phycobiliproteins, with potential applications in biotechnology. The study highlighted the suitability of *Nostoc* sp. 136 for laboratory cultivation and suggested avenues for further research, particularly in exploring biomass composition and bioactivities.

**Abstract:**

Cyanobacteria, photoautotrophic Gram-negative bacteria, play a crucial role in aquatic and terrestrial environments, contributing significantly to fundamental ecological processes and displaying potential for various biotechnological applications. It is, therefore, critical to identify viable strains for aquaculture and establish accurate culture parameters to ensure an extensive biomass supply for biotechnology purposes. This study aims to establish optimal laboratory batch culture conditions for *Nostoc* 136, sourced from Alga_2_O, Coimbra, Portugal. Preliminary investigations were conducted to identify the optimal culture parameters and to perform biomass analysis, including protein and pigment content. The highest growth was achieved with an initial inoculum concentration of 1 g.L^−1^, using modified BG11 supplemented with nitrogen, resulting in a Specific Growth Rate (SGR) of 0.232 ± 0.017 μ.day^−1^. When exposed to white, red, and blue LED light, the most favourable growth occurred under a combination of white and red LED light exhibiting an SGR of 0.142 ± 0.020 μ.day^−1^. The protein content was determined to be 10.80 ± 2.09%. Regarding the pigments, phycocyanin reached a concentration of 200.29 ± 30.07 µg.mL^−1^, phycoerythrin 148.29 ± 26.74 µg.mL^−1^, and allophycocyanin 10.69 ± 6.07 µg.mL^−1^. This study underscores the influence of light and nutrient supplementation on the growth of the *Nostoc* biomass.

## 1. Introduction

Cyanobacteria were the first oxygen-producing photosynthetic organisms on Earth. They emerged about 3.5-billion years ago and are still thriving today. Their significant contributions to oxygen, organic carbon, and nitrogen cycles, in various habitats, emphasise their wide distribution due to metabolic versatility and structural conservation. Among cyanobacteria, those that are equipped with nitrogenase play a key role in fixing atmospheric nitrogen by converting it to ammonia through biological nitrogen fixation [1,2]. This process not only produces organic nitrogen, making it available to other photosynthetic organisms, but it also leads to the production of secondary metabolites that promote plant growth and resistance to stress, with benefits for soil fertility and crop productivity, among other features.

Recognising cyanobacteria as versatile cell factories, scientific research has explored their ability to produce high-value products such as primary metabolites (carbohydrates, proteins, lipids), but also secondary metabolites such as mycosporine-like amino acids and phytohormones, among others. These compounds have been applied as food supplements, cosmetics, pharmaceuticals, biomaterials, biofuels, bioremediation, and agriculture [3,4,5,6,7,8,9,10]. Thus, cyanobacterial farming has emerged as a potential solution to increase the availability of biomass for biotechnological purposes. Despite these advances in scientific research, nonetheless, the economic viability of using cyanobacteria requires careful analysis. Overcoming economic barriers involves producing value-added chemicals to offset the overall processing costs [11].

Among the most promising cyanobacteria is the genus *Nostoc*, a heterocystous filamentous cyanobacterium. Heterocysts develop as a response to nitrogen deprivation, particularly in the absence of ammonium from the environment or from the nutrient medium [12]. Akinetes, on the other hand, differentiate under diazotrophic conditions, i.e., in the absence of nitrogen, and are influenced by extracellular metabolites [13]. The genus also stands out for the formation of unbranched trichomes embedded in a dense exopolysaccharides (EPS) sheath. This sheath not only provides moisture to the colony but also exhibits metal-concentrating properties, contributing to its resilience against external threats [14]. The UV-absorbing pigments within the EPS, such as scytonemin and mycosporin-like amino acids, further shield *Nostoc* against solar radiation [15]. *Nostoc* is also recognised for its ability to efficiently produce cyanophycin, a nitrogen-storage compound [16], as well as soil improvement [17] and plant-growth enhancement [18].

Since *Nostoc* is recognised for its environmental and biotechnological applications, over the past decades, research has been geared towards finding the optimum conditions for its production in aquaculture. Studies on nutrient supply [19,20], photoautotrophic, mixotrophic, and heterotrophic cultivation [21], light intensity and quality [22], and the type of bioreactor used [23,24,25], among others, have produced valuable knowledge on the cultivation of different *Nostoc* species. Other authors have focused on the production of bioactive compounds, namely the exopolysaccharides [26,27,28], phycobiliproteins [29,30], cyanophycin [16], polysaccharides [31], and biodegradable polymers [32,33], as well as mycosporine-like amino acids [34]. Cultivation trials also focused on specific applications, including food production [35], bioremediation [14,36], and biodiesel production [37].

The aim of this work was to grow a strain of heterocystous cyanobacteria, *Nostoc* sp. 136, obtained from the Alga_2_O bank, in Coimbra, Portugal, under different conditions. These trials sought to study cultivation conditions (density, nutrients, light) in order to optimize *Nostoc* growth. These trials offer insights into the biomass production potential of *Nostoc* sp. 136 under controlled laboratory settings, allowing us to understand if the strain has the required physiological characteristics to be efficiently grown in laboratory conditions.

## 2. Materials and Methods

**Figure 1:**
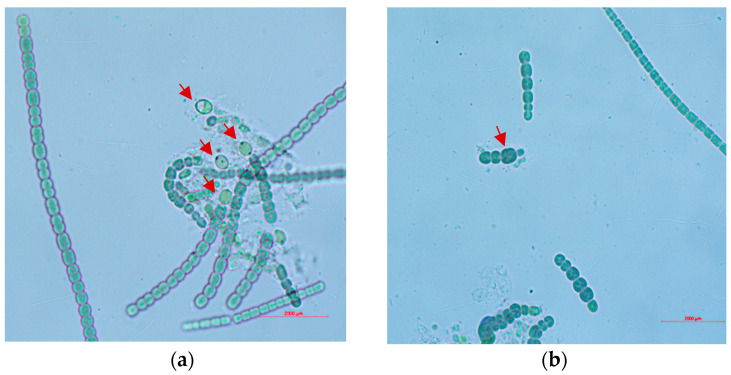
Photomicrographs of *Nostoc* 136 strain, showing oval intercalary heterocysts with two polar nodules (**a**) and enlarged akinete (**b**) (red arrows), larger than vegetative cells.

The cyanobacterial strain *Nostoc* sp. 136 (Figure 1), was obtained from the Alga_2_O Collection, located in Coimbra, Portugal. The strain was kept in a climatic room (20 ± 2 °C), with constant aeration, under a white LED light of 8–10 μmol m^−2^ s^−1^, with a photoperiod set at 16 h:8 h (Light:Dark), supplied with BG11 medium prepared according to McFadden and Melkonian [38,39].

### 2.1. Culture Conditions

The *Nostoc* sp. 136 culture was kept in 250 mL volumetric flasks in batch mode with constant aeration, filtered through a 20 μm filter (Sartorius Stedim Biotech GmbH, Goettingen, Germany). Due to the extreme heterogeneity of *Nostoc* biomass, which tends to form aggregates of trichomes, it is not possible to homogenise the culture for regular sampling, so the biomass was only measured at the end of each trial.

Also, due to biomass heterogeneity, the starter culture of the inoculum was centrifuged at 2000× *g* for 3 min, allowing the biomass to settle. Then, an equal weight of *Nostoc* biomass was added to each flask containing 200 mL of modified BG11 medium (mBG11).

**Figure 2:**
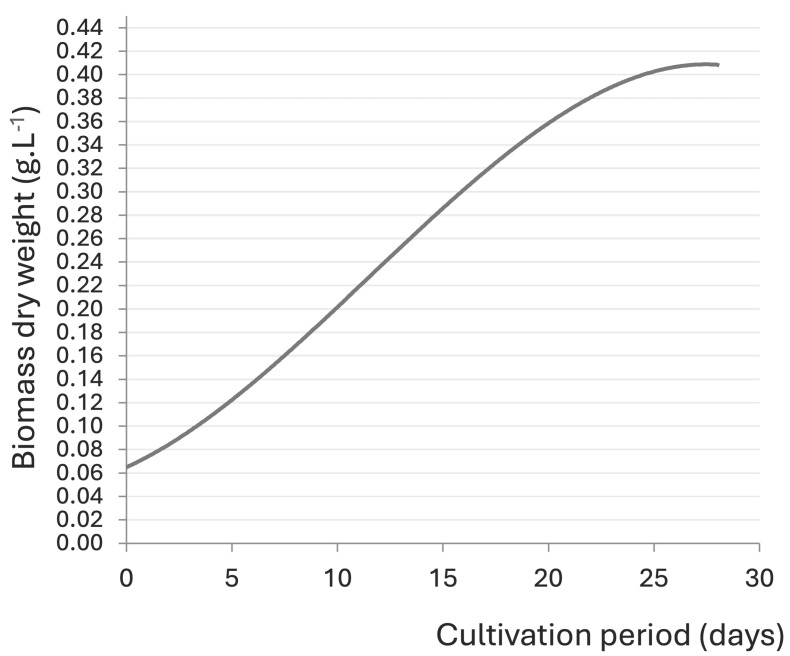
Growth curve of *Nostoc* sp. 136, under cultivation for 30 days. Adapted from Mouga et al. [40].

This *Nostoc* strain submitted to cultivation exhibits a classic growth-curve shape, tending to grow slowly. Thus, the exponential phase is reached after 12 to 15 days of cultivation, as shown in Figure 2 [40]. Therefore, all assays lasted for 15 days.

The algae growth rate was measured as specific growth rate (SGR) (μ.day^−1^), and it was calculated according to Lavasseur et al. [41]:

μ = (ln C_2_ − ln C_1_)/(t_2_ − t_1_) (1)

Biomass productivity (yield) was expressed on a volumetric basis (g.L^−1^.day^−1^) and calculated according to the following formula [37]:

P = C_2_ − C_1_/Δt (2)

where,

C_1_—biomass concentration at time t_1_, C_2_—biomass concentration at time t_2_, Δt—difference in time.

As stated above, due to *Nostoc* sp. 136 biomass heterogeneity, it is not possible to assess it quantitatively using optical density. Therefore, the fresh weight and dry weight of the sample, in mg.L^−1^, were evaluated. Likewise, the initial inoculum was calculated based on centrifuged biomass and fresh weight.

The first test was performed to determine the best initial concentration using MBG11 as the nutrient medium (Trial 1).

In Trial 2, two commercial media were analysed in addition to mBG11: Nutribloom^®^ (Phytobloom by Necton, Belamandil, Portugal) and FloraNova^®^ Grow (General Hidroponics, Hawthorne Gardening Co., Vancouver, BC, Canada). The former medium has shown remarkable results in the growth of microalgae [42,43,44]. The latter medium has been developed for the growth of plants in hydroponics and showed good results growing *Arthrospira platensis* [45]. The concentration used for the three media is the recommended concentration for mBG11 regarding the macronutrient nitrate (150 g.L^−1^). Thus, 10 mL.L^−1^ of mBG11, 0.25 mL.L^−1^ of FloraNova, and 1.25 mL.L^−1^ of Nutribloom were used (the media composition of all three media is in Supplementary Material Table S1).

In this trial, it was established that the medium that promotes the strongest growth of *Nostoc* 136 is mBG11. This medium was, hence, used in subsequent trials.

In the third trial, some of the nutrients in mBG11 were manipulated to assess their impact on the growth of *Nostoc*. The macronutrient nitrogen was assayed due to its influence on the synthesis of biomolecules such as proteins and, therefore, phycobilins. Also, the trace elements, iron and magnesium, were evaluated; the first because it is involved in nitrogen fixation and is part of the chemical composition of the nitrogenase enzyme, and the last due to its presence in the chlorophyll and Rubisco, as well as in the production of ATP. The nutrient concentrations used are provided in Table 1.

Finally, in Trial 4, the effect of different light wavelengths was assessed. The control for this trial was the mBG11 medium, with a cool-white LED and an irradiance of 8.3 µmol m^−2^ s^−1^. The irradiance was maintained between tests, with only the combination of wavelengths changed. For this purpose, programmable LED (USB RGB 5050, Romwish LED, Shengwei Lighting Co., Dong Guan, China) lights were used and organised in three horizontal rows, 2 cm apart, at the height of the culture flask [46,47]. Red and blue light were tested, as well as a combination of White+Blue and White+Red, allowing for different wavelengths to be tested, as shown in Table 1.

### 2.2. Biochemical Analysis

Protein extraction was conducted using the protocol adapted from Parimi et al. [48]. A 100 mL dry sample was centrifuged (5810R, Eppenford, Madrid, Espanha) at 8000× *g* for 15 min at room temperature. The supernatant was discarded, and the pellet was dried in an oven at 60 °C overnight. Protein extraction was conducted by solubilising the samples in 1 M HCl and centrifuging at 8670× *g* for 35 min. The supernatant was analysed using the Pierce BCA protein assay kit (Thermo Scientific, Waltham, MA, USA) as in Martin et al. [49]. Absorbance of the final samples was measured on a spectrophotometer (Evolution 201, Thermo Scientific, Waltham, MA, USA) at 652 nm.

As to the quantification of phycobiliproteins, one gram of fresh biomass, previously frozen at −18 °C, was submitted to three freeze–thaw cycles of 12 h each, protected from light [29]. The biomass was then centrifuged at 5000× *g* for 10 min at room temperature. The supernatant was scanned between 400 and 900 nm in a spectrophotometer. The phycobilin content was calculated using the equations developed by Bennett and Bogorad [50]:

PC [mg/mL] = (OD(615 nm) − 0.474 × OD(652 nm))/5.34(3)

APC [mg/mL] = (OD(652 nm) − 0.208 × OD(615 nm))/5.09(4)

PE [mg/mL] = (OD(562 nm) −2.41 × [PC] − 0.849 [APC])/9.62(5)

where,

[PC]—C-Phycocyanin
[APC]—Allophycocyanin
[PE]—C-Phycoerythrin
OD(615 nm)—Absorbance of the sample at wavelength of 615 nm
OD(652 nm)—Absorbance of the sample at wavelength of 652 nm
OD(562 nm)—Absorbance of the sample at wavelength of 562 nm

### 2.3. Statistical Analysis

All trials were conducted in triplicate and the data are expressed as mean ± standard deviation. Statistical analyses were considered significant at a level of 5% (*p*-value < 0.05). To test normality and variance homogeneity, the Kolmogorov–Smirnov and Shapiro–Wilk tests were used, respectively. As the data met the assumptions, the one-way ANOVA test was used. Statistical analyses were performed using IBM SPSS statistical software, Version 27.0 (IBM Corporation, Armonk, NY, USA).

## 3. Results

### 3.1. Culture Conditions

**Figure 3:**
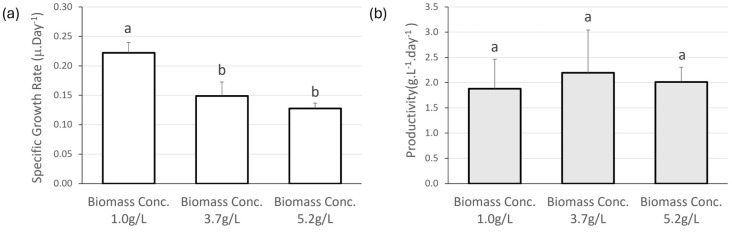
(**a**) Specific Growth Rate (μ.day^−1^) and (**b**) productivity (g.L^−1^.day^−1^) of *Nostoc* sp. 136, with three biomass concentrations (1.0 g.L^−1^, 3.7 g.L^−1^ and 5.2 g.L^−1^). Values are presented as mean ± SD (*n* = 3). Different lower-case letters indicate statistically significant differences (*p*-value < 0.05) in the one-way ANOVA.

The first trial was performed to assess the optimal initial biomass concentration of *Nostoc* that would provide the best growth rate. Three different concentrations were evaluated, the lowest of which gave the highest specific growth rate (0.222 ± 0.018 μ.day^−1^), significantly higher than the other two concentrations (*p*-value < 0.05). As to the productivity, the highest value was registered for the initial biomass concentration of 3.7 g.L^−1^, with 2.195 ± 0.847 g.L^−1^.day^−1^. However, there is no statistical difference between the three concentrations (Figure 3).

Although there was no correlation between SGR and productivity, the SGR showed statistically significant differences for the initial concentration of 1 g.L^−1^, and, therefore, it was decided to use this concentration in subsequent trials.

**Figure 4:**
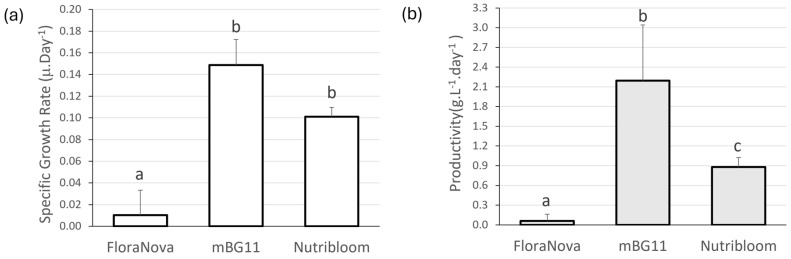
(**a**) Specific Growth Rate (μ.day^−1^) and (**b**) productivity (g.L^−1^.day^−1^) of *Nostoc* sp. 136 grown under three different nutrient media (FloraNova, mBG11, and Nutribloom). Values are presented as mean ± SD (*n* = 3). Different lower-case letters indicate statistically significant differences (*p*-value < 0.05) in the one-way ANOVA.

The second trial analysed three different nutrient media, two of which were commercially available, to assess if these could be used to efficiently grow *Nostoc*, instead of mBG11, which is expensive, time-consuming, and labour-intensive to prepare. Figure 4 shows the SGR and productivity obtained for this trial. *Nostoc* shows a significantly higher growth rate in mBG11 (0.149 ± 0.0237 μ.day^−1^) and in Nutribloom (0.1010 ± 0.009 μ.day^−1^) than in FloraNova (0.010 ± 0.0229 μ.day^−1^). The productivity is in line with the data obtained for SGR, with mBG11 inducing higher *Nostoc* yields than Nutribloom (2.195 ± 0.847 and 0.879 ± 0.147 g.L^−1^.day^−1^, respectively) and the latter showing much higher *Nostoc* yields than FloraNova (0.058 ± 0.101 g.L^−1^.day^−1^), always with statistically significant differences between groups. Hence, mBG11 was used in the following trials.

**Figure 5:**
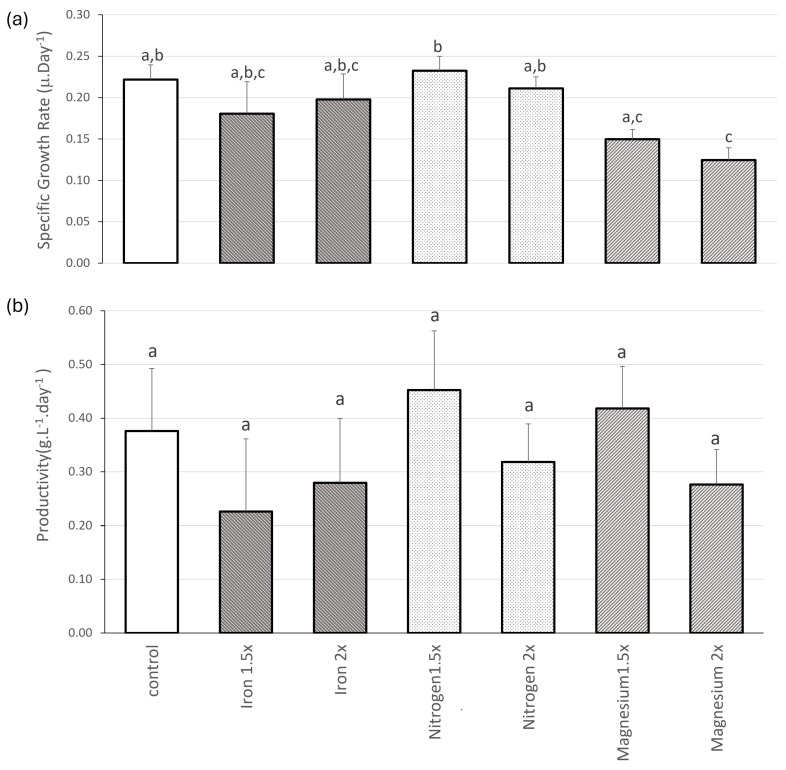
(**a**) Specific Growth Rate (μ.day^−1^) and (**b**) productivity (g.L^−1^.day^−1^) of *Nostoc* sp. 136 grown under different medium supplementation (control, iron 1.5×, iron 2×, nitrogen 1.5×, nitrogen 2×, magnesium 1.5×, and magnesium 2×). Values are presented as mean ± SD (*n* = 3). Different lower-case letters indicate statistically significant differences (*p*-value < 0.05) in the one-way ANOVA.

The third trial evaluated the impact of medium supplementation on the growth rate and productivity. As can be seen in Figure 5, only the 1.5-fold supplementation of nitrogen increased the growth rate of *Nostoc* when compared to the control, but without statistical significance (0.232 ± 0.018 μ.day^−1^ and 0.222 ± 0.017 μ.day^−1^, respectively). All other supplements produced lower growth rates, with magnesium supplementation, either 1.5 times or two times, producing significantly lower *Nostoc* growth rates than the highest growth rate (0.149 ± 0.012 and 0.125 ± 0.015 μ.day^−1^, respectively).

When analysing productivity, no statistically significant differences were observed between the supplementation group and the control. Nevertheless, certain supplements exhibited high productivity in comparison to the control, specifically nitrogen at 1.5 times and magnesium at 1.5 times the standard concentration (0.376 ± 0.167, 0.452 ± 0.111, and 0.418 ± 0.0782 g.L^−1^.day^−1^, respectively). Notably, increased supplementation, such as doubling the nutrient concentration compared to the control, resulted in decreased productivity (iron, nitrogen, and magnesium at two times presented productivity of 0.279 ± 0.120, 0.318 ± 0.071, and 0.277 ± 0.065 g.L^−1^.day^−1^, respectively). This suggests a trend where higher nutrient concentrations may lead to toxicity within the organisms, thereby compromising productivity.

As no significantly higher growth rates or productivities were found between the control and supplemented media, the final test was conducted with standard mBG11.

**Figure 6:**
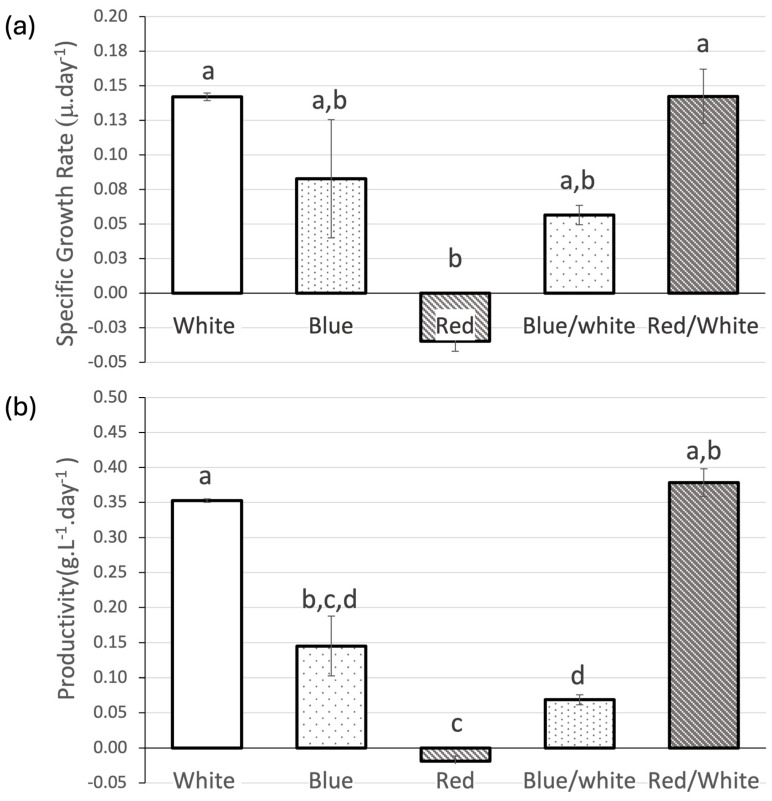
(**a**) Specific Growth Rate (μ.day^−1^) and (**b**) productivity (g.L^−1^.day^−1^) of *Nostoc* sp. 136 grown under different wavelengths (control—white LED, Blue LED, Red LED, White–Blue LED combo, White–Red LED combo). Values are presented as mean ± SD (*n* = 3). Different lower-case letters indicate statistically significant differences (*p*-value < 0.05) in the one-way ANOVA.

In the fourth trial, different light wavelengths were evaluated while keeping irradiance constant. The control (white LED light) presented an SGR of 0.1420 ± 0.003 μ.day^−1^. The best growth was obtained with a white–red LED combo, with 0.1424 ± 0.019 μ.day^−1^, while red LED light showed negative growth, with an SGR of −0.0348 ± 0.007 μ.day^−1^, being statistically different from the first two. Blue LED light produced intermediate values, with no statistical differences (Figure 6). Once again, high productivity is promoted by the red–white LED combination (0.379 ± 0.112 g.L^−1^.day^−1^), though with no statistical difference compared to the control (0.353 ± 0.0.018 g.L^−1^.day^−1^). However, both these cultures show significantly higher yields when compared to the other *Nostoc* cultures under different wavelengths. The red LED light caused the worst *Nostoc* yield presenting a negative value (−0.019 ± 0.003 g.L^−1^.day^−1^).

### 3.2. Biochemical Analysis

The protein and phycobilin contents were assessed in *Nostoc* grown in mBG11 medium and with white LED light. The protein content recorded for the *Nostoc* biomass at the end of the trial was 10.803 ± 2.089% (DW).

**Figure 7:**
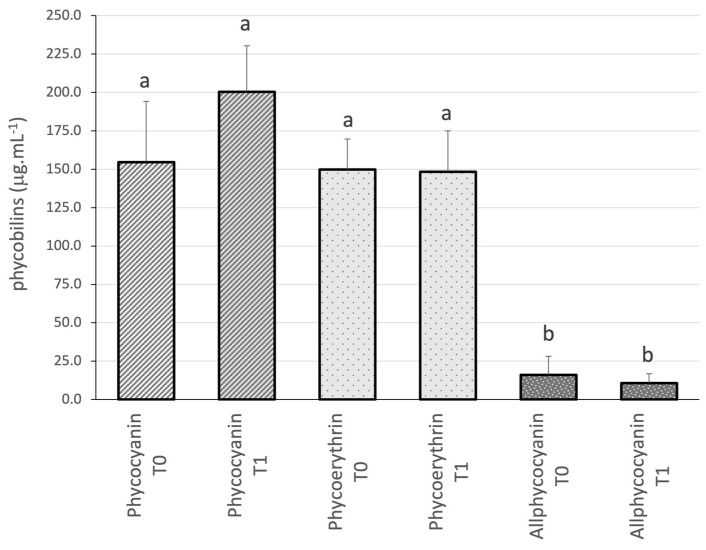
Phycobilin content (μg.mL^−1^) of *Nostoc* sp. 136 grown under white LED light at the beginning (T0) and end (T1) of a 15-day trial. Values are presented as mean ± SD (*n* = 3). Different lower-case letters indicate statistically significant differences (*p*-value < 0.05) in the one-way ANOVA.

As to the phycobilins, they were measured before and after the 15-day trial (Figure 7). *Nostoc* 136 shows a similar concentration of phycocyanin and phycoerythrin at the beginning of the trial, with 154.655 ± 39.544 μg.mL^−1^ and 148.292 ± 26.735 μg.mL^−1^, respectively. As to allophycocyanin, as expected, the concentration was significantly lower at the beginning of the trial (16.052 ± 12.118 μg.mL^−1^). At the end of the trial, phycocyanin increased to its maximum concentration (200.293 ± 30.074 μg.mL^−1^), while phycoerythrin remained unchanged and allophycocyanin was slightly reduced. None of these fluctuations are statistically significant.

## 4. Discussion

Cultivating *Nostoc* offers numerous environmental benefits, producing biomass for use in soil stabilization, nutrient cycling, pollution remediation, and ecosystem restoration [14,17,36,51,52,53]. *Nostoc* also produces a wide array of bioactive compounds with potential pharmaceutical, nutraceutical, and cosmeceutical applications. These include antioxidants, antimicrobial agents, anticancer, antiviral, anti-inflammatory compounds, UV-absorbing pigments, prebiotic peptides, and polysaccharides with immunomodulatory properties [27,37,54,55]. Thus, cultivating *Nostoc* under optimized conditions allows for the scalable production of these bioactive compounds, which can be further developed into functional ingredients for various biotechnological industries. These trials have provided new information on the ability of *Nostoc* sp. 136 to be cultivated in laboratory conditions.

The growth rate of most cyanobacteria is relatively low (0.1–0.5 μ.day^−1^) [56], and *Nostoc* sp. 136 is no exception. The specific growth rate achieved in our study surpassed that reported by Yu et al. [21] for *Nostoc flagelliforme* (0.12 μ.day^−1^) in a phototrophic culture, yet it fell short of the rates observed by Baracho [57] in the cultivation of *Nostoc* CCIBt 3248 (0.3 μ.day^−1^) and *Nostoc* CCIBt 3249 (0.7 μ.day^−1^).

The first trial evaluated the initial biomass concentration of the culture, a pivotal parameter deserving optimization. Whilst dense cultures offer potential benefits such as volume efficiency and reduced energy demands, it is crucial to note that high-density cultivation has been linked with diminished productivity [58]. The density of the starter culture exerted a noticeable influence on the growth rate and productivity of *Nostoc* sp. 136 growth rate and productivity after a 15-day period. Remarkably, the lowest initial concentration (1 g.L^−1^) allowed a significantly higher growth rate (0.222 ± 0.018 μ.day^−1^), mirroring the findings of Van Khanh et al. [59] in their study on *Arthrospira platensis*. This is likely to be attributed to light utilization efficiency. As the density of the cyanobacterial culture rises, there is a corresponding increase in biomass production rates. However, beyond a certain threshold, the culture’s density becomes so elevated that the specific growth rate declines due to increased self-shadowing, leading to a reduction in available light [60]. Consequently, the need to grow dense cultures must be balanced with the provision of sufficient light to sustain optimal growth rates.

The second trial evaluated different culture media on the growth of *Nostoc* sp. 136, trying to substitute the commonly used laboratory medium BG11. BG11 medium has been widely reported as a mainstream medium for cyanobacterial biomass and lipid production, especially for freshwater microalgae [20]. This medium is composed of the macronutrients and trace elements required for the growth and metabolism of cyanobacteria. Nitrogen and phosphorus are the elements that are most important for microalgal metabolism. Without nitrogen, both photosynthetic rates and oxygen-production rates decrease, significantly affecting cell growth and pigment production, including both chlorophyll and phycobiliproteins [30,61]. Cyanobacteria can use both organic and inorganic nitrogen dissolved in water, due to the presence of heterocysts and nitrogenase. Nitrogenase consists of two components, an iron protein, and an iron–molybdenum protein [1]. Cyanobacteria also require magnesium chelatases (for chlorophyll), in addition to iron-chelatases (haem) present in the antenna-like domain [62]. Thus, iron, magnesium, and molybdenum are, thus, three of the most important trace elements. In addition to these three, most organisms also need Cu, Zn, Mn, Ca, and K, which contribute to the bulk ions and are key to the synthesis of various macromolecules, such as enzymes and transmembrane transporters [62].

We investigated the appropriateness of FloraNova and Nutribloom to grow *Nostoc* sp. 136 since BG11 is not suitable for large-scale cultivation due to its labour-intensive preparation. As stated before, both commercial media showed adequate results in the growth of other microalgae, including *A. platensis* [43,44,46]. The Nutribloom medium proved to be promising for growing *Nostoc*. The growth rate and productivity were lower than that of *Nostoc* grown on BG11, although the growth rate was not significant. This shows that the chemical composition of the two media, both macronutrients and trace elements, is suitable for the nutritional needs of *Nostoc*, allowing the species to metabolise and grow adequately [20]. FloraNova, nonetheless, led to a significantly lower growth rate and productivity, demonstrating that the nutrient content is unbalanced in relation to *Nostoc*’s requirements. It is undeniable that this medium lacks several trace elements (Zn, Mo, Cu, Co, Mg) that play important roles in cyanobacterial metabolism. Thus, unlike *A. platensis* [46], the lack of such nutrients prevented *Nostoc* from thriving.

In the third trial, using the mBG11 medium as a control, we increased the concentration of nitrate, iron, and magnesium in the medium to assess the impact of these three nutrients on the growth and productivity of the cyanobacterium. Increasing nitrate 1.5 times increased both the growth rate and productivity of *Nostoc*, but with no statistical differences. Our results are in contrast to the finding of Lee et al. [30], who found that nitrogen supplementation did not improve cyanobacterial growth. Trentin and co-workers [16] also found a decrease in growth rate with nitrate supplementation. However, these authors assessed inorganic nitrogen. According to these authors, cyanobacteria produce cyanophycin as a temporary nitrogen reserve compound. In heterocystous cyanobacteria such as *Nostoc*, the accumulation of cyanophycin is correlated to a peak of nitrogenase activity, and the accumulation of cyanophycin is higher in the absence of inorganic nitrogen than in the presence of a nitrogen source, which tends to suppress the formation of heterocysts.

The source of nitrogen for the growth of cyanobacteria found in all the media tested is nitrate, an organic form of nitrogen, which is reduced to nitrite and then to ammonium to be finally incorporated into the storage components. Therefore, as Cottas et al. stated, there is a slight increase in *Nostoc* growth with nitrate supplementation [19]. Nitrogen shortages, on the other hand, are very detrimental to the cell, causing it to utilise a portion of the nitrogen present in phycobiliproteins, which also function as the cell’s nitrogen storage structures. Reducing organic nitrogen availability, hence, results in a decrease in these pigments in the cell, while supplementing the culture media with nitrogen tends to favour the production of phycobiliproteins [19].

As for the supplementation of magnesium, which is an important trace element since it is part of important molecules such as enzymes and pigments, we expected to increase the photosynthetic rate and, therefore, the growth rate. The slight increase in Nostoc, grown at 1.5 times the magnesium concentration, may be due to the ability of magnesium sulphate (MgSO_4_) to induce heterocyst formation [63]. As also noticed by Qiong–Che et al. studying *Monoraphidium,* Mg^2+^ may play a role in cellular processes and metabolic pathways related to growth and lipid accumulation in this microalgae species [64].

As to iron, it is a growth-promoting factor [65]. Iron deficiency in cyanobacteria leads to a decrease in chlorophyll *a* content, a reduction in cell diameter, a depletion of the electron transport chain, and a decrease in photosynthesis and respiration processes [66]. In contrast, high iron levels in the culture medium tend to increase chlorophyll *a*, total cell volume, and oxygen-production rate, resulting in a higher nitrogen-fixation rate and photosynthesis [67]. Thus, due to the role of iron in cyanobacterial physiology, it was expected that iron supplementation would lead to an increase in the growth of *Nostoc*. However, neither SGR nor productivity was improved. This may be explained by an excess of free intracellular iron, which is detrimental because it catalyses the formation of reactive oxygen species (ROS). In particular, H_2_O_2_ reacts with ferrous iron to produce the highly reactive hydroxyl radical. The damage caused by oxidation depends on the rigorous control of iron homeostasis [68]. Therefore, it seems that the supplementation may have induced some degree of oxidative stress that decreased cell growth.

Since it is well recognised that light is a key factor in regulating the growth and metabolism of cyanobacteria [69], the final trial determined the impact of different wavelengths of light on cell growth. Cyanobacteria usually do not endure light intensities exceeding 100 μmol photons.m^−2^ s^−1^. To counteract photoinhibition, they reduce chlorophyll content while maintaining a relatively constant level of carotenoids, which serve as effective photoprotectors. As light intensity increases, photosynthetic efficiency declines due to both photosaturation and photoinhibition [16]. In addition to irradiance, the colour of the light also impacts the performance of cyanobacteria, showing that, among other factors, the wavelength can influence the accumulation of biomass and metabolites. Red LED light was found to promote the highest cell growth and cell densities, whereas blue LED light stimulated the accumulation of nitrogen compounds in the form of phycobiliproteins at the expense of cell growth [70]. Our results determined that the best growth occurred with a combination of white and red light, both for SGR and productivity, although not significantly. Pagels et al. [71] reported that blue-light supplementation showed little or no improvements to the culture of *Cyanobium* sp., and red light did not improve growth but triggered the production of lipids, phycocyanin, carotenoids, and total antioxidant compounds. Our findings regarding blue light align closely with those of Pagels et al. suggesting that blue-supplemented light is not used as an active energy source for photosynthesis. Conversely, red light may enhance cyanobacterial cell growth by inducing mRNA expression of psaE, a photosynthesis-related gene [72], and it often stimulates the production of bioactive compounds, including carotenoids, fatty acids, phenolic compounds, and phycocyanin [68,71]. Hence, our findings diverge from those of these authors. This discrepancy could be attributed to the conditions tested (wavelength and irradiance) where, due to photoinhibition, respiratory metabolism might surpass photosynthesis, resulting in cell death [22].

As to protein content, the protein level found by Baracho et al. [57] for *Nostoc* is higher, ranging from 26.2% to 54.7%. The different extraction methods used by these authors (optimised sequential extraction in trichloroacetic acid and NaOH) followed Slocombe et al. [73] method, which has been shown to be more effective than the one used in this work.

Lastly, concerning the phycobiliprotein content, *Nostoc* sp. 136 showed interesting levels of both phycocyanin and phycoerythrin under white LED light. These values exceeded those reported by Lee [30] under identical conditions. As emphasized by these authors, the manipulation of culture conditions notably augments pigment production, deserving further exploration in subsequent studies.

## 5. Conclusions

This research paper underscores the suitability of cultivating *Nostoc* sp. 136 under laboratory conditions. The strain has been shown to adapt well to laboratory settings and respond favourably to various treatments, including changes in nutrient supplementation and different light conditions. Further research is necessary to ascertain whether the biomass composition, beyond proteins and phycobiliproteins, holds promise, and whether the biomass demonstrates bioactivities. However, existing data on similar species suggest that this strain may hold potential for further exploration. Moreover, the species’ heterogeneity arises from its high content of exopolysaccharides, as highlighted in our previous work [40], compounds that deserve to be further investigated.

Furthermore, cultivating *Nostoc* in thin-layer raceway ponds, where light can penetrate deeper into the culture layer, may be required in industrial units. These ponds offer a favourable surface area-to-volume ratio and facilitate rapid light-/dark-cell cycling, thereby enhancing photosynthetic efficiency [23]. They also provide a simple means of maintaining the buoyancy of *Nostoc*, which otherwise tends to form aggregates and settle.

## Figures and Tables

**Table 1:** Summary of the conditions of the experimental trials performed (*n* = 3).

Trial 1. Inoculum Concentration				
Inoculum concentration	mBG11 (1.0 g.L^−1^)	mBG11 (3.7 g.L^−1^)		mBG11 (5.2 g.L^−1^)
Trial 2. Medium selection				
Medium	mBG11 (10 mL.L^−1^)	Nutribloom (1.25 mL.L^−1^)		FloraNova (0.25 mL.L^−1^)
Trial 3. Nutrients supplementation				
Nitrogen	1× (1.5000 g.L^−1^)	1.5× (2.2500 g.L^−1^)		2× (3.0000 g.L^−1^)
Iron	1× (0.0030 g.L^−1^)	1.5× (0.0045 g.L^−1^)		2× (0.0060 g.L^−1^)
Magnesium	1× (0.0750 g.L^−1^)	1.5× (0.1125 g.L^−1^)		2× (0.1500 g.L^−1^)
Trial 4. Light conditions				
Single LED	Blue—peak at 465 nm	White (450 and 550–620 nm)		Red—peak at 635 nm
Combined LED	White and Blue (440–470 + 550–620 nm)		White and red (550–640 nm)	

## Data Availability

Data are contained within the article.

## Supplementary Materials

The following supporting information can be downloaded at: https://www.mdpi.com/article/10.3390/biology13050306/s1, Table S1: Nutrient media composition (mBG11, NutriBloom, and FloraNova Grow).

## References

[1] Berman-Frank I., Lundgren P., Falkowski P. (2003). Nitrogen Fixation and Photosynthetic Oxygen Evolution in Cyanobacteria. Res. Microbiol..

[2] Rucker H.R., Kaçar B. (2023). Enigmatic Evolution of Microbial Nitrogen Fixation: Insights from Earth’s Past. Trends Microbiol..

[3] Enzing C., Ploeg M., Barbosa M., Sijtsma L. (2014). Microalgae-Based Products for the Food and Feed Sector: An Outlook for Europe.

[4] Flores C., Tamagnini P. (2019). Looking Outwards: Isolation of Cyanobacterial Released Carbohydrate Polymers and Proteins. J. Vis. Exp..

[5] Ruiz J., Olivieri G., de Vree J., Bosma R., Willems P., Reith J.H., Eppink M.H.M., Kleinegris D.M.M., Wijffels R.H., Barbosa M.J. (2016). Towards Industrial Products from Microalgae. Energy Environ. Sci..

[6] Santini G., Biondi N., Rodolfi L., Tredici M.R. (2021). Plant Biostimulants from Cyanobacteria: An Emerging Strategy to Improve Yields and Sustainability in Agriculture. Plants.

[7] Thanuja G., Ramasamy A., Karthikeyan S., Arumugam M., Kathiresan S., Subramani N. (2020). Microalgae and cyanobacteria: Role and applications in agriculture. Applied Algal Biotechnology.

[8] Laroche C. (2022). Exopolysaccharides from Microalgae and Cyanobacteria: Diversity of Strains, Production Strategies, and Applications. Mar. Drugs.

[9] Liang F., Englund E., Lindberg P., Lindblad P. (2018). Engineered Cyanobacteria with Enhanced Growth Show Increased Ethanol Production and Higher Biofuel to Biomass Ratio. Metab. Eng..

[10] Kumar J., Singh T.D.M.B., Kumar A., Mishra A.K., Tiwari D.N., Rai A.N. (2019). Cyanobacteria: Applications in Biotechnology. Cyanobacteria from Basic Science to Applications.

[11] Kondi V., Sabbani V., Alluri R., Karumuri T.S.K., Chawla P., Dasarapu S., Tiwari O.N. (2022). Cyanobacteria as Potential Bio Resources for Multifaceted Sustainable Utilization. New and Future Developments in Microbial Biotechnology and Bioengineering.

[12] Christman H.D., Campbell E.L., Meeks J.C. (2011). Global Transcription Profiles of the Nitrogen Stress Response Resulting in Heterocyst or Hormogonium Development in *Nostoc punctiforme*. J. Bacteriol..

[13] Zhao J., Peter Wolk C., Whitworth D.E. (2007). Developmental Biology of Heterocysts. Myxobacteria: Multicellularity and Differentiation.

[14] Ahad R.I.A., Goswami S., Syiem M.B. (2017). Biosorption and Equilibrium Isotherms Study of Cadmium Removal by *Nostoc muscorum* Meg 1: Morphological, Physiological and Biochemical Alterations. 3 Biotech.

[15] Whitton B.A., Potts M. (2002). The Ecology of Cyanobacteria.

[16] Trentin G., Piazza F., Carletti M., Zorin B., Khozin-Goldberg I., Bertucco A., Sforza E. (2023). Fixing N2 into Cyanophycin: Continuous Cultivation of *Nostoc* sp. PCC 7120. Appl. Microbiol. Biotechnol..

[17] Gheda S.F., Ahmed D.A. (2015). Improved Soil Characteristics and Wheat Germination as Influenced by Inoculation of *Nostoc kihlmani* and *Anabaena cylindrica*. Rend. Lincei.

[18] Kollmen J., Strieth D. (2022). The Beneficial Effects of Cyanobacterial Co-Culture on Plant Growth. Life.

[19] Cottas A.G., Teixeira T.A., Cunha W.R., Ribeiro E.J., de Souza Ferreira J. (2022). Effect of Glucose and Sodium Nitrate on the Cultivation of *Nostoc* sp. PCC 7423 and Production of Phycobiliproteins. Braz. J. Chem. Eng..

[20] Touloupakis E., Zittelli G.C., Benavides A.M.S., Torzillo G. (2022). Growth and Photosynthetic Performance of *Nostoc linckia* (Formerly *N. calcicola*) Cells Grown in BG11 and BG110 Media. Photochem. Photobiol. Sci..

[21] Yu H., Jia S., Dai Y. (2009). Growth Characteristics of the Cyanobacterium *Nostoc flagelliforme* in Photoautotrophic, Mixotrophic and Heterotrophic Cultivation. J. Appl. Phycol..

[22] Ma R., Lu F., Bi Y., Hu Z. (2015). Effects of Light Intensity and Quality on Phycobiliprotein Accumulation in the Cyanobacterium *Nostoc sphaeroides* Kützing. Biotechnol. Lett..

[23] Celis-Plá P.S.M., Rearte T.A., Neori A., Masojídek J., Bonomi-Barufi J., Álvarez-Gómez F., Ranglová K., Carmo da Silva J., Abdala R., Gómez C. (2021). A New Approach for Cultivating the Cyanobacterium *Nostoc calcicola* (MACC-612) to Produce Biomass and Bioactive Compounds Using a Thin-Layer Raceway Pond. Algal Res..

[24] Walther J., Erdmann N., Stoffel M., Wastian K., Schwarz A., Strieth D., Muffler K., Ulber R. (2022). Passively Immobilized Cyanobacteria *Nostoc* species BB 92.2 in a Moving Bed Photobioreactor (MBPBR): Design, Cultivation, and Characterization. Biotechnol. Bioeng..

[25] Strieth D., Weber A., Robert J., Stiefelmaier J., Kollmen J., Volkmar M., Lakatos M., Jordan V., Muffler K., Ulber R. (2021). Characterization of an Aerosol-Based Photobioreactor for Cultivation of Phototrophic Biofilms. Life.

[26] Fischer D., Schlösser U.G., Pohl P. (1997). Exopolysaccharide Production by Cyanobacteria Grown in Closed Photobioreactors and Immobilized Using White Cotton Towelling. J. Appl. Phycol..

[27] Tiwari O.N., Khangembam R., Shamjetshabam M., Sharma A.S., Oinam G., Brand J.J. (2015). Characterization and Optimization of Bioflocculant Exopolysaccharide Production by Cyanobacteria *Nostoc* sp. BTA97 and *Anabaena* sp. BTA990 in Culture Conditions. Appl. Biochem. Biotechnol..

[28] Cui L., Xu H., Zhu Z., Gao X. (2017). The Effects of the Exopolysaccharide and Growth Rate on the Morphogenesis of the Terrestrial Filamentous Cyanobacterium *Nostoc flagelliforme*. Biol. Open.

[29] Reis A., Mendes A., Lobo-Fernandes H., Empis J.A., Novais J.M. (1998). Production, Extraction and Purification of Phycobiliproteins from *Nostoc* sp.. Bioresour. Technol..

[30] Lee N.K., Oh H.M., Kim H.S., Ahn C.Y. (2017). Higher Production of C-Phycocyanin by Nitrogen-Free (Diazotrophic) Cultivation of *Nostoc* sp. NK and Simplified Extraction by Dark-Cold Shock. Bioresour. Technol..

[31] Shen S.G., Jia S.R., Wu Y.K., Yan R.R., Lin Y.H., Zhao D.X., Han P.P. (2018). Effect of culture conditions on the physicochemical properties and antioxidant activities of polysaccharides from *Nostoc flagelliforme*. Carbohydr. Polym..

[32] Bhati R., Mallick N. (2012). Production and Characterization of Poly(3-Hydroxybutyrate-Co-3-Hydroxyvalerate) Co-Polymer by a N2-Fixing Cyanobacterium, *Nostoc muscorum* Agardh. J. Chem. Technol. Biotechnol..

[33] Sharma L., Mallick N. (2005). Accumulation of Poly-β-Hydroxybutyrate in *Nostoc muscorum*: Regulation by PH, Light–Dark Cycles, N and P Status and Carbon Sources. Bioresour. Technol..

[34] Roshan S.K., Farhangi M., Emtyazjoo M., Rabbani M. (2015). Effects of Solar Radiation on Pigmentation and Induction of a Mycosporine-like Amino Acid in Two Cyanobacteria, *Anabaena* sp. and *Nostoc* sp. ISC26. Eur. J. Phycol..

[35] Feng Y.-N., Zhang Z.-C., Feng J.-L., Qiu B.-S. (2012). Effects of UV-B Radiation and Periodic Desiccation on the Morphogenesis of the Edible Terrestrial Cyanobacterium *Nostoc flagelliforme*. Appl. Environ. Microbiol..

[36] El-Sheekh M.M., El-Shouny W.A., Osman M.E.H., El-Gammal E.W.E. (2005). Growth and Heavy Metals Removal Efficiency of *Nostoc muscorum* and *Anabaena subcylindrica* in Sewage and Industrial Wastewater Effluents. Environ. Toxicol. Pharmacol..

[37] El Shafay S.M., Gaber A., Alsanie W.F., Elshobary M.E. (2021). Influence of Nutrient Manipulation on Growth and Biochemical Constituent in *Anabaena variabilis* and *Nostoc muscorum* to Enhance Biodiesel Production. Sustainability.

[38] McFadden G.I., Melkonian M. (1986). Use of Hepes Buffer for Microalgal Culture Media and Fixation for Electron Microscopy. Phycologia.

[39] Rippka R., Deruelles J., Waterbury J.B., Herdman M., Stanier R.Y. (1979). Generic Assignments, Strain Histories and Properties of Pure Cultures of Cyanobacteria. J. Gen. Microbiol..

[40] Mouga T., Simões F., Moreira V., Martins A., Ferreira C., Ramos R., Afonso C. (2023). Producing Cyanobacteria to Use as Biostimulants. Proceedings of the 2nd International Conference on Water Energy Food and Sustainability (ICoWEFS 2022).

[41] Levasseur M., Thompson P.A., Harrison P.J. (1993). Physiological Acclimation of Marine Phytoplankton to Different Nitrogen Sources. J. Phycol..

[42] Bastos C.R.V., Maia I.B., Pereira H., Navalho J., Varela J.C.S. (2022). Optimisation of Biomass Production and Nutritional Value of Two Marine Diatoms (Bacillariophyceae), *Skeletonema costatum* and *Chaetoceros calcitrans*. Biology.

[43] Olofsson M., Lamela T., Nilsson E., Bergé J.P., del Pino V., Uronen P., Legrand C. (2012). Seasonal Variation of Lipids and Fatty Acids of the Microalgae *Nannochloropsis oculata* Grown in Outdoor Large-Scale Photobioreactors. Energies.

[44] Concórdio-Reis P., Cardeira M., Macedo A.C., Ferreira S.S., Serra A.T., Coimbra M.A., Amorim A., Reis M.A.M., Freitas F. (2023). Novel Exopolysaccharide Produced by the Marine Dinoflagellate *Heterocapsa* AC210: Production, Characterization, and Biological Properties. Algal Res..

[45] Carneiro J., Gomes S., Freitas M., Afonso C., Mouga T. (2018). Growth of *Arthrospira platensis* under laboratory and outdoor conditions: Assessment of the effects of light and different nutrient media. Front. Mar. Sci..

[46] Wishkerman A., Wishkerman E. (2017). Application Note: A Novel Low-Cost Open-Source LED System for Microalgae Cultivation. Comput. Electron. Agric..

[47] Davidson M.W. Fundamentals of Light-Emitting Diodes. Zeiss Microscopy 2008, 1–12. https://zeiss-campus.magnet.fsu.edu/print/lightsources/leds-print.html.

[48] Parimi N.S., Singh M., Kastner J.R., Das K.C., Forsberg L.S., Azadi P. (2015). Optimization of Protein Extraction from *Spirulina platensis* to Generate a Potential Co-Product and a Biofuel Feedstock with Reduced Nitrogen Content. Front. Energy Res..

[49] Martin I., Cabán-Hernández K., Figueroa-Santiago O., Espino A.M. (2015). *Fasciola hepatica* Fatty Acid Binding Protein Inhibits TLR4 Activation and Suppresses the Inflammatory Cytokines Induced by Lipopolysaccharide In Vitro and In Vivo. J. Immunol..

[50] Bennett A., Bogorad L. (1973). Complementary Chromatic Adaptation in a Filamentous Blue-Green Alga. J. Cell Biol..

[51] Sholkamy E.N., El-Komy H., Al-Arfaj A.A., Abdel-Megeed A., Mostafa A.A. (2012). Potential Role of *Nostoc muscorum* and *Nostoc rivulare* as Biofertilizers for the Enhancement of Maize Growth under Different Doses of N-Fertilizer. Afr. J. Microbiol. Res..

[52] Maqubela M.P., Mnkeni P.N.S., Issa O.M., Pardo M.T., D’Acqui L.P. (2009). Nostoc Cyanobacterial Inoculation in South African Agricultural Soils Enhances Soil Structure, Fertility, and Maize Growth. Plant Soil.

[53] Chai Y., Cai P., Gillor O., Kuramae E.E., A Costa O.Y., Raaijmakers J.M. (2018). Microbial Extracellular Polymeric Substances: Ecological Function and Impact on Soil Aggregation. Front. Microbiol..

[54] Sánchez-Bayo A., Morales V., Rodríguez R., Vicente G., Bautista L.F. (2020). Cultivation of Microalgae and Cyanobacteria: Effect of Operating Conditions on Growth and Biomass Composition. Molecules.

[55] Khalifa S.A.M., Shedid E.S., Saied E.M., Jassbi A.R., Jamebozorgi F.H., Rateb M.E., Du M., Abdel-Daim M.M., Kai G.-Y., Al-Hammady M.A.M. (2021). Cyanobacteria—From the Oceans to the Potential Biotechnological and Biomedical Applications. Mar. Drugs.

[56] Chojnacka K., Noworyta A. (2004). Evaluation of *Spirulina* sp. Growth in Photoautotrophic, Heterotrophic and Mixotrophic Cultures. Enzym. Microb. Technol..

[57] Baracho D.H., Lombardi A.T. (2023). Study of the Growth and Biochemical Composition of 20 Species of Cyanobacteria Cultured in Cylindrical Photobioreactors. Microb. Cell Fact..

[58] Straka L., Rittmann B.E. (2018). Effect of Culture Density on Biomass Production and Light Utilization Efficiency of *Synechocystis* sp. PCC 6803. Biotechnol. Bioeng..

[59] Van Khanh N., Thi Diem N., Tuyet Nhan L.T., Cu P.V., Khanh Van T.Q., Thi Hoan N.T. (2017). The Effects of Nutritional Media and Initial Cell Density on the Growth and Development of *Spirulina platensis*. J. Agric. Sci. Technol. A.

[60] Hewes C.D. (2015). Not All Culture Is Created Equal: A Comparative Study in Search of the Most Productive Cultivation Methodology. Algal Res..

[61] Aranda-Vega Y., Bhatt P., Huang J.-Y., Brown P., Bhasin A., Hussain A.S., Simsek H. (2024). Biodegradability and Bioavailability of Dissolved Substances in Aquaculture Effluent: Performance of Indigenous Bacteria, Cyanobacteria, and Green Microalgae. Environ. Pollut..

[62] Cavet J.S., Borrelly G.P.M., Robinson N.J. (2003). Zn, Cu and Co in Cyanobacteria: Selective Control of Metal Availability. FEMS Microbiol. Rev..

[63] Dawar S., Mohanty P., Behera B.K. (1999). Sustainable Hydrogen Production in the Cyanobacterium *Nostoc* sp. ARM 411 Grown in Fructose- and Magnesium Sulphate-Enriched Culture. World J. Microbiol. Biotechnol..

[64] Che R.Q., Wang Q.M., Huang L., Zhao P., Yu X.Y. (2014). Effects of Additional Mg^2+^ on the Growth and Lipid Accumulation of *Monoraphidium* sp. FXY-10 under Mixotrophic Conditions. Adv. Mater. Res..

[65] El-Sheekh M.M., Prášil O., El-Mohsnawy E. (2021). Physiological and Spectroscopical Changes of the Thermophilic Cyanobacterium *Synechococcus elongatus* under Iron Stress and Recovery Culture. Acta Physiol. Plant..

[66] Halac S.R., Ruibal-Conti A.L., Mengo L.d.V., Ullmer F., Cativa A., Bazan R., Rodriguez M.I. (2023). Effect of Iron Availability on the Growth and Microcystin Content of Natural Populations of *Microcystis* spp. from Reservoirs in Central Argentina: A Microcosm Experiment Approach. Phycology.

[67] Rueter J.G., Ohki K., Fujita Y. (1990). The Effect of Iron Nutrition on Photosynthesis and Nitrogen Fixation in Cultures of *Trichodesmium* (Cyanophyceae). J. Phycol..

[68] Latifi A., Ruiz M., Zhang C.-C. (2009). Oxidative Stress in Cyanobacteria. FEMS Microbiol. Rev..

[69] Mohanty B., Majedi S.M., Pavagadhi S., Te S.H., Boo C.Y., Gin K.Y.H., Swarup S. (2022). Effects of Light and Temperature on the Metabolic Profiling of Two Habitat-Dependent Bloom-Forming Cyanobacteria. Metabolites.

[70] Jung C.H.G., Waldeck P., Sykora S., Braune S., Petrick I., Küpper J.H., Jung F. (2022). Influence of Different Light-Emitting Diode Colors on Growth and Phycobiliprotein Generation of *Arthrospira platensis*. Life.

[71] Pagels F., Bonomi-Barufi J., Vega J., Abdala-Díaz R., Vasconcelos V., Guedes A.C., Figueroa F.L. (2020). Light Quality Triggers Biochemical Modulation of *Cyanobium* sp.—Photobiology as Tool for Biotechnological Optimization. J. Appl. Phycol..

[72] Kim N.N., Shin H.S., Park H.G., Lee J., Kil G.S., Choi C.Y. (2014). Profiles of Photosynthetic Pigment Accumulation and Expression of Photosynthesis-Related Genes in the Marine Cyanobacteria *Synechococcus* sp.: Effects of LED Wavelengths. Biotechnol. Bioprocess Eng..

[73] Slocombe S.P., Ross M., Thomas N., McNeill S., Stanley M.S. (2013). A Rapid and General Method for Measurement of Protein in Micro-Algal Biomass. Bioresour. Technol..

