# Supplementary material for: Unveiling the Cultivation of Nostoc sp. under Controlled Laboratory Conditions
Source: Biology (Basel). 2024 Apr 28;13(5):306. doi: 10.3390/biology13050306 (PMC11118237; doi:10.3390/biology13050306)
Supplement: Supplementary file 1 [file biology-13-00306-s001.zip › biology-2957628-supplementary.pdf]

biology MDPI

Table S1. Chemical composition of the media used in the trials.

**BG11 modified**	**Nutribloom** (Necton S.A.)	**Flora Nova Grow** (General Hydroponics)
$NaNO_3$	$NaNO_3$	N (NH_4^+ and NO_3^-)
KH_2PO_4	KH_2PO_4	P_2O_5
$CaCl_2.2H_2O$	$ZnCl_2$	K_2O
$ZnSO_4.7H_2O$	$ZnSO_4$	SO_4
$MnCl_2.4H_2O$	$MnCl_2$	CaO
$Na_2MoO_4.2H_2O$	$Na_2MoO_4.2H_2O$	MgO
$CoCl_2.7H_2O$	$CoCl_2.6H_2O$	Fe
$CuSO_4.5H_2O$	$CuSO_4.5H_2O$	
Na_2EDTA	EDTA	
$MgSO_4.7H_20$	$MgSO_4$	
$FeSO_4$	$FeCl_3$	
Citric Acid	Thiamine	
H_3BO_3	Biotin	
	Vitamin B12	
